# Paraboloid Structured Silicon Surface for Enhanced Light Absorption: Experimental and Simulative Investigations

**DOI:** 10.1186/s11671-015-1087-9

**Published:** 2015-09-29

**Authors:** Firoz Khan, Seong-Ho Baek, Jasmeet Kaur, Imran Fareed, Abdul Mobin, Jae Hyun Kim

**Affiliations:** Division of Nano and Energy Convergence Research, Daegu Gyeongbuk Institute of Science & Technology (DGIST), 50-1 Sang-Ri, Hyeonpung-Myeon, Dalseong-gun, Daegu, 711-873 Republic of Korea; Physics of Energy Harvesting Division, CSIR-National Physical Laboratory, Dr. K.S. Krishnan Marg, New Delhi, 110 012 India; Department of Computer Science, Jamia Millia Islamia, New Delhi, 110 025 India

**Keywords:** Solar cells, Surface modification, Reflectance, Light trapping, Paraboloid structure

## Abstract

In this paper, we present an optical model that simulates the light trapping and scattering effects of a paraboloid texture surface first time. This model was experimentally verified by measuring the reflectance values of the periodically textured silicon (Si) surface with the shape of a paraboloid under different conditions. A paraboloid texture surface was obtained by electrochemical etching Si in the solution of hydrofluoric acid, dimethylsulfoxide (DMSO), and deionized (DI) water. The paraboloid texture surface has the advantage of giving a lower reflectance value than the hemispherical, random pyramidal, and regular pyramidal texture surfaces. In the case of parabola, the light can be concentrated in the direction of the Si surface compared to the hemispherical, random pyramidal, and regular pyramidal textured surfaces. Furthermore, in a paraboloid textured surface, there can be a maximum value of 4 or even more by anisotropic etching duration compared to the hemispherical or pyramidal textured surfaces which have a maximum *h*/*D* (depth and diameter of the texture) value of 0.5. The reflectance values were found to be strongly dependent on the *h*/*D* ratio of the texture surface. The measured reflectance values were well matched with the simulated ones. The minimum reflectance value of ~4 % was obtained at a wavelength of 600 nm for an *h*/*D* ratio of 3.75. The simulation results showed that the reflectance value for the *h*/*D* ratio can be reduced to ~0.5 % by reducing the separations among the textures. This periodic paraboloidal structure can be applied to the surface texturing technique by substituting with a conventional pyramid textured surface or moth-eye antireflection coating.

## Background

The cost of the solar energy can be reduced by enhancing solar cell efficiency using some advanced technology to fabricate solar cells with reduced losses [1, 2], namely, reflection [3], recombination [4], and resistive losses [5, 6]. The surface texturing of the wafers is an important part of the silicon (Si) solar cell’s fabrication process in R&D and production levels [7]. This process can be utilized to enhance the absorption of light due to enhancing the back surface reflectance and path length of light and reduction in front surface reflection. This process also encourages the carrier collection near the junction.

For a bare Si surface, the reflection loss is around ~30 % due to reflection of light from the front surface resulting in a small amount of absorption which yields poor efficiency. To reduce these losses, several techniques have been invented. Out of all the methods created and invented, surface texturing has been widely used to reduce the reflection losses. It reduces the front surface reflection, increases the internal reflections, and also increases the light path lengths inside the cell due to the overall light absorption increasing. Several surface texturing methods, like mechanical grooving [8], reactive ion etching [9], laser texturing [10], alkaline [7], and acidic texturing [11] have been used to increase the absorption of light inside the solar cell. Alkaline and acidic texturing has been widely used for the commercial production of Si wafer-based solar cells.

Alkaline anisotropic etchants like sodium hydroxide (NaOH) and potassium hydroxide (KOH) at low concentrations and temperatures of 80–90 °C can form square base pyramidal structures randomly distributed over the cell surface. The reflectance value of ~12 % was obtained at a wavelength of 600 nm for the random pyramidal structure [12]. Baek et al. [13] also made a regular pyramidal structure using electrochemical etching. In a regular pyramidal structure, the reflectance value is reduced to ~10 %. The pyramidal structures allow for multiple reflections and hence trap any incoming ray light and reduce the reflection.

Several attempts have been made in previous studies to explain the variation of the reflectance values by taking scanning electron microscopy (SEM) images of the texture surface and theoretical modeling of reflectance [7, 14–17]. Previous studies [7, 14–17] have assumed that the acid textured surfaces are a part of the hemisphere, and also, the variation of reflectance with a *h*/*D* (depth and diameter of the texture) ratio was also determined. In case of the texture surface as a part of the hemisphere, the minimum reflectance value of ~15 % can be achieved for a maximum achievable value of an *h*/*D* ratio of 0.5.

Macdonals et al. [14] developed a masked reactive ion etching (RIE) method, which is better than the wet acid, maskless reactive ion etching method. Xi et al. [18] observed a part of hemispherical structure in acid texturization and calculated that reflectivity at 500 nm start declining when the *h*/*D* exceeds 0.29.

Several researchers have introduced analytical models for Si nano-wire mats [19], and nano-holes [20]. Kim and Kim [21] used ultrasonic vibration during alkaline texturing of multicrystalline Si (mc-Si) to improve uniformity. Gangopadhyay et al. [22] obtained 14.0–14.5 % efficiency in an industrial production line with a yield >95 % by using mc-Si substrates of large size and textured them in a solution of NaOH-NaOCl at 80 °C to restrict the step height to <5 μm. Si nano-structures have been characterized using various techniques [23, 24]. Tsujino et al. [25] used metallic catalysts for acid texturization. Hazeland and Hu [26] formed a titled pyramidal structure by using potassium carbonate, which shows a lower reflectance compared to polished surfaces. Campbell et al. [27] suggested that top surface reflection could be eliminated by using a tilted pyramidal structure. Wang et al. [28] wire textured the mc-Si wafers by using a self-assembled mask. Houser et al. [29] used nano-imprint lithography for solar cell texturization, whereas Xu et al. [30] developed lithography-free sub 100 nm nano-cone array antireflection layers for the application of solar cells. Houser et al. [29] found that the texture surface is a part of paraboloids. It was found that acid textured surfaces are not always part of the hemisphere but also have a parabolic structure. The paraboloid structure has more potential to reduce the reflection loss.

In this work, we investigated the Si texture surface as a part of paraboloid and developed a model for surface reflection of paraboloid texture surfaces. This model was used to predict the reflectance as a function of wavelength and *h*/*D* ratio for the paraboloid textured Si surface. This model considers the reflection from the entire solar spectrum, bulk absorption, and the contribution of the rear surface to light trapping. In the case of parabola, there is more possibility that the light could be concentrated inside the Si solar cell compared to the hemispherical, random pyramidal, and regular pyramidal textured surfaces. For hemispherical or pyramidal textured surfaces, the maximum *h*/*D* value can reach 0.5, whereas in paraboloid textured surfaces, the maximum h/D value can reach a maximum of 4 or even more by anisotropic chemical etching.

### Modeling of Parabolic Textured Surface

**Fig. 1:**
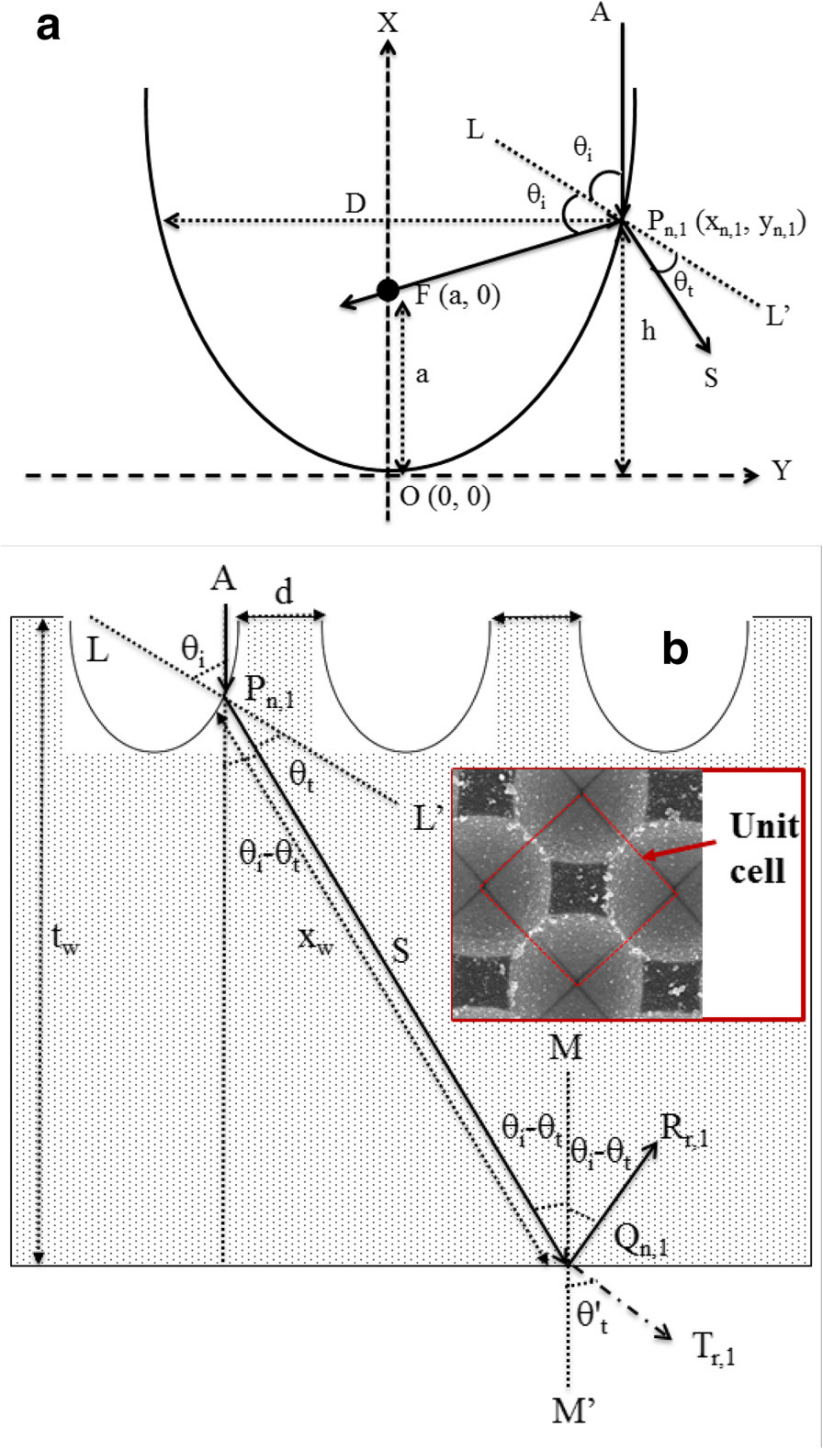
Schematic of ray tracing (**a**) in the paraboloid structure at front surface and (**b**) in the bulk Si and at rear surface (in the *inset*, *top view* of unit cell of SEM that used for simulation)

For simulation, we assumed that the textured surface is part of a paraboloid (axis in *x*-direction) with vertex at O (0, 0) and focus F (a, 0). The schematics of ray tracing from the paraboloid structured front surface and rear surface (plane) are shown in Fig. 1a, b, respectively. The top view of unit cell of SEM that used for simulation is shown in the inset of Fig. 1b. A beam of light is incident along AP direction with making an incident angle *θ*_i_ with the normal (LL’) to the textured surface. The incident beam split into two parts after strike at point *P*_*n*,1_ (generally say P). The reflected part moves along PF direction (making an angle *θ*_i_ with LL’), while the refracted beam goes in PS direction (making an angle *θ*_t_, with LL’). Let assume the height of P from the bottom is *h* and diameter of circle passing through point P is *D*.

The values of *x* and *y* are replaced by *D*/2 and *h* in the equation of parabola. With this equation, we can get a relation

1 $$ {D}^2=4.4a\cdot h $$

where *D*, *h*, and 4*a* denote the width, depth, and latus rectum of the paraboloid textured structure, respectively.

The calculations were done using a ray tracing model. Divide the half width of the texture into *N* points. Let these points be *P*_*n*,1_(*x*_*n*,1_, *y*_*n*,1_), where *n* = 0, 1,….*N*. From Eq. (1), *x*_*n*,1_, *y*_*n*,1_ can be found as

2 $$ {y}_{n,1}=n\times \frac{D}{2N}\kern2.25em n=0,1,\dots, N $$

3 $$ {x}_{n,1}=\frac{{y^2}_{n,1}}{4a} $$

*P*_*n*,2_, *P*_*n*,3_ , …., *P*_*n*,*m*_ are the points of reflection that correspond to the light incident on point *P*_*n*,1_, where *m* is the number of times the light hits the textured surface. The relationship between these points can be found by the rotation matrix:

4 $$ \left(\begin{array}{c}\hfill {x}_{m,n+1}\hfill \\ {}\hfill {y}_{m,n+1}\hfill \end{array}\right)\kern0.5em =\kern0.5em \left(\begin{array}{c}\hfill cos\phi - sin\phi \hfill \\ {}\hfill sin\phi \kern1.5em  cos\phi \hfill \end{array}\right)\left(\begin{array}{c}\hfill {x}_{m,n}\hfill \\ {}\hfill {y}_{m,n}\hfill \end{array}\right) $$

where *φ* is − (*π* − 2*θ*).

It is assumed that incident light is directed normally (at 90°) to the textured parabolic surface (normal to wafer surface). Due to the properties of the parabolic mirror, all the reflected light will pass through the focus *F* of the parabolic surface. The reflectance *R*_*n*,1_ for the single ray that the incident on *P*_*n*,1_ can be calculated as [15]

5 $$ {R}_{n,1}=\frac{R_{\mathrm{TE}n,1}+{R}_{\mathrm{TM}n,1}}{2} $$

6 $$ {R}_n={\left[\frac{1}{2}\left(\frac{sin^2\left({\theta}_i-{\theta}_t\right)}{sin^2\left({\theta}_i+{\theta}_t\right)}+\frac{tan^2\left({\theta}_i-{\theta}_t\right)}{tan^2\left({\theta}_i+{\theta}_t\right)}\right)\right]}^{k_m} $$

where *R*_TE*n*,1_ and *R*_TM*n*,1_ denote the polarized reflectance of light on the TE and TM modes, respectively. The reflectance for the *m*th annular surface element in terms of incident angle *θ*_i_ and refracted angle *θ*_t_ angles is given by Eqs. (7) and (8)

7 $$ {\theta}_{\mathrm{i}}={cos}^{-1}\left(\frac{a}{\sqrt{a^2+ ax}}\right) $$

8 $$ {\theta}_{\mathrm{t}}={sin}^{-1}\left(\frac{sin\left({\theta}_{\mathrm{i}}\right)}{\mu}\right) $$

The values *θ*_i_ and *θ*_t_ lie between 0° and 90°.

Here, *k*_n_ is the no. of reflection (no. of times the light reflected back on the surface, which is an incident on point *P*_*n*,1_).

It can be seen that for a lower wavelength, there is no rear surface reflection effect, as the light gets absorbed due to a high absorption coefficient for a lower wavelength. However, for a higher wavelength, the value of *R*_r_ has a huge effect on the total reflection. The refracted light along direction PS incident on back surface at Q making an angle (*θ*_i_–*θ*_t_) with normal (MM’) to the rear surface as shown in Fig. 1b. This incident beam PS on rare surface again split into two parts, first reflected one along QR direction making the same angle (*θ*_i_–*θ*_t_) with MM’, whereas transmitted beam goes along QT direction (making angle *θ*_t_’ with MM’).

The total reflectance can increase due to a lower value of absorption coefficient and more rear surface reflection. The rear surface reflection (*R*_r_) was calculated using Eq. (9) [31]

9 $$ {R}_{\mathrm{r}}=\left[\frac{1}{2}\left(\frac{sin^2\left\{\left({\theta}_{\mathrm{i}}-{\theta}_{\mathrm{t}}\right)-{\theta}_{\mathrm{t}}^{\hbox{'}}\right\}}{sin^2\left\{\left({\theta}_{\mathrm{i}}-{\theta}_{\mathrm{t}}\right)+{\theta}_{\mathrm{t}}^{\hbox{'}}\right\}}+\frac{tan^2\left\{\left({\theta}_{\mathrm{i}}-{\theta}_{\mathrm{t}}\right)-{\theta}_{\mathrm{t}}^{\hbox{'}}\right\}}{tan^2\left\{\left({\theta}_{\mathrm{i}}-{\theta}_{\mathrm{t}}\right)+{\theta}_{\mathrm{t}}^{\hbox{'}}\right\}}\right)\right] $$

10 $$ {\theta}_{\mathrm{t}}^{\hbox{'}}={sin}^{-1}\left(\mu \kern.03em .\kern.05em  sin\left({\theta}_{\mathrm{i}}-\theta \mathrm{t}\right)\right) $$

The attenuation factor (*A*_m_) via absorption of bulk Si can be calculated as [16]

11 $$ {A}_{\mathrm{m}}={\displaystyle {e}^{-\alpha {x}_w}} $$

12 $$ {x}_w\approx \raisebox{1ex}{${t}_w$}\!\left/ \!\raisebox{-1ex}{$\mathrm{C}\mathrm{o}\mathrm{s}\left({\displaystyle {\theta}_i}-{\displaystyle {\theta}_t}\right)$}\right. $$

Here, h << *t*_w_, *t*_w_ is the wafer thickness and *x*_w_ is the path length traveled by the refracted light inside the Si wafer, which make an angle of (*θ*_i_–*θ*_t_) with the normal (MM’) of rare plane surface. As the light pass through the wafer, its attenuation factor increases hence probability of light gets absorbed within the wafer increases and reflectivity decreases.

For (*θ*_i_–*θ*_t_) ≥ critical angle, total internal reflection will occur.

The overall reflection after absorption and rear surface reflection can be expressed as [16]

13 $$ {R}_{n,\mathrm{t}} = {R}_n + \left(1\hbox{-} {R}_n\right){R}_{\mathrm{r}}\left(1\hbox{-} {R}_{\mathrm{r}}\right){A^2}_m+\left(1\hbox{-} {R}_n\right){R}_{\mathrm{r}}\left(1\hbox{-} {R}_{\mathrm{r}}\right){A^4}_m+...... $$

The total reflection from the front surface is the average reflection of these weighted points by their surface area given as

14 $$ R={\displaystyle \sum_{n=0}^{N+1}\frac{R_{n,t}\times d{S}_n}{S}} $$

where *dS*_*n*_ is the small annular surface element area and *S* is the front cross-sectional area of the paraboloid structure and can be calculated as

15 $$ d{S}_n=\pi \left({\displaystyle {y}_{n,1}^2}-{\displaystyle {y}_{{}_{n+1,1}}^2}\right) $$

16 $$ S=\pi {\displaystyle {\left(\frac{D}{2}\right)}^2} $$

The Eqs. (1)–(16) were used to calculate the theoretical values of the reflectance of paraboloid front texture surfaces.

## Methods

Si paraboloid micro-pore arrays were prepared on B-doped p-type <100> Si wafers with a resistivity of 1~10 Ωcm and wafer thickness (*t*_w_) of 550 μm by electrochemical etching method. In order to make Si pore arrays with a parabolic shape, we first oxidized the wafers to get a silicon oxide (SiO_2_) mask using thermal oxidation. A square pattern of 2 μm × 2 μm size was obtained on SiO_2_ using photolithography. The patterned samples were dipped in a KOH etchant at 80 °C to make inverse-shaped pyramidal notches that would act as regions for concentrating an electrical bias. The electrochemical etching was performed in a solution of hydrofluoric acid, dimethylsulfoxide (DMSO), and deionized (DI) water. A thin aluminum layer was deposited on the backside of the wafer to produce an Ohmic contact between the Si wafer and working electrode by DC magnetron sputtering method. The electrochemical etching was performed under a constant current density mode of different biases in a Teflon bath. A platinum wire was used as a counter electrode. The Si wafer with Al back contact acted as a working electrode. The sample area exposed to the electrolyte solution was approximately 2 cm^2^. The etch rate was approximately 1.1~1.3 μm/min, and the pore length was controlled by etching time under otherwise nominally identical conditions.

Field emission scanning electron microscopy (FE-SEM) images of the front and cross-sectional view of paraboloid textured Si surfaces were taken under ×5000 magnification using the Hitachi FE-SEM, Model S-4800. The reflectance measurements were carried out with a wavelength range of 300–1200 nm using the UV-Vis-NIR spectrophotometer, Model Cary-5000.

## Results and Discussion

Figure 1 shows the schematic of ray tracing in the parabolic structure instead of the paraboloid for picture clarity. In this diagram, the axis of parabola is assumed along the *x*-axis. A ray falls along the axis of parabola. Similar to the parabolic mirror, the ray incident on any point *P*_*n*,1_ (*x*_*n*,1_, *y*_*n*,1_) of the inner surface of the parabola will pass through the focal point *F*. The vertex *O* of the parabola is assumed as origin (0, 0). The distance of point *P* from the *y*-axis is *h*, which is known as height or depth of textures, and at the same point, the diameter of the circular ring is *D*. The distance between points *F* and *O* is *a*. Hence, the latus rectum of the parabola is 4*a* (as taken in the theoretical section for calculations). When a ray is incident on point *P* at an incident angle, *θ*_i_ is split into two parts. The first reflected part goes in the air along the PF direction while making the same reflected angle as *θ*_i_. Another refracted part goes along the PS direction while making an angle *θ*_t_.

**Fig. 2:**
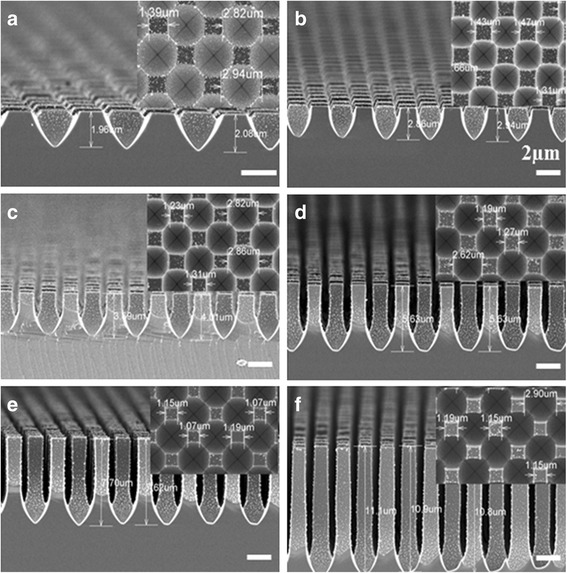
Front and cross-sectional (in the *inset*) view of SEM images of paraboloid texture silicon surfaces for etching duration (**a**) 95, (**b**) 140, (**c**) 185, (**d**) 300, (**e**) 390 and (**f**) 575 s

**Fig. 3:**
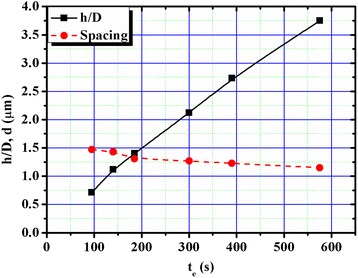
Variation of *h/D* ratio and spacing between two adjacent paraboloid textures with etching duration at 25 °C

Figure 2a–f shows the FE-SEM micrographs of paraboloid textured surfaces for etching durations (*t*_e_) of 95, 140, 185, 300, 390 and 575 s, respectively. The *h* and *D* values were calculated from the SEM images of the paraboloid structures, as shown in Fig. 2. A slight increase in *D* value is observed ~2.63 μm for *t*_e_ = 95 s to ~2.90 μm for *t*_e_ = 575 s, while the *h* values changed very fast from ~1.87 μm to ~10.86 μm. The obtained *h/D* ratios for the samples a, b, c, d, e, and f were 0.71, 1.12, 1.40, 2.12, 2.73, and 3.75, respectively. However, the corresponding spacing between two adjacent paraboloid textures (*d*) are 1.37, 1.34, 1.32, 1.27, 1.18, 1.11 μm, respectively (as listed in Table 1). The *h/D* along with *d* vs. *t*_e_ are plotted in Fig. 3. It can be seen that *h*/*D* is increased with increase in *t*_e_. However, the rate of change in *h/D* ratio decreased with time from its initial value of 0.009/s to 0.005/s due to a decrease in the etching rate along (100) direction. Thus, the *d* value is decreased from 1.47 μm (for *t*_e_ = 95 s) to 1.15 μm (for *t*_e_ = 575 s). The *d* and *D* values have been used to calculated the coverage area of untexture (*a*_1_) and texture (*a*_2_) surfaces. The *a*_1_/*a*_2_ ratio is also decreased with *t*_e_ as illustrated in Table 1.

**Table 1:** The *h*/*D* values along with surface area ratio of untexture (*a*_1_) to texture (*a*_2_) with etching duration (*t*_*e*_)

Etching duration (s)	*h*/*D*	Spacing between textures (*d*, μm)	*a*_1_/*a*_2_
95	0.71	1.37	0.271
140	1.12	1.34	0.253
185	1.40	1.32	0.242
300	2.12	1.27	0.216
390	2.73	1.18	0.175
575	3.75	1.11	0.144

**Fig. 4:**
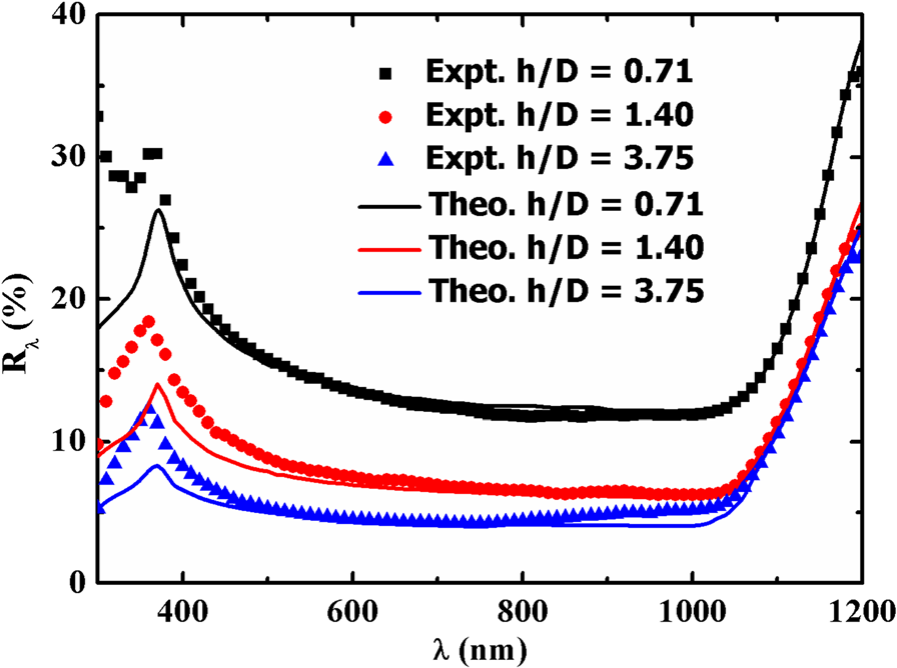
Experimental and theoretical reflectance spectra for *h/D* values of 0.71, 1.40, and 3.75 for spacing values of 1.37, 1.34, and 1.11, respectively

The experimental measured and simulated reflectance (*R*_*λ*_) values vs. the wavelength (*λ*) for three different *h/D* ratios (0.71, 1.40, and 3.75) are shown in Fig. 4. The corresponding *a*_1_/*a*_2_ values used for simulation of *R*_*λ*_ are 0.271, 0.253, and 0.144 for *h*/*D* = 0.71, 1.40, and 3.75, respectively. It can be seen that the simulated reflectance curve for all *h/D* ratios matched well with their experimental values in the wavelength range of 450–1200 nm. The simulated values of *R*_*λ*_ are slightly lower than the experimental *R*_*λ*_ values for the wavelength lower than 450 nm. The minimum *R*_*λ*_ value of ~4 % was obtained at a wavelength of 600 nm for the *h/D* ratio of ~3.75. It can be noted that in the case of part of the hemisphere surface, the minimum reflectance value of ~15 % was obtained for the maximum achievable value of the *h/D* ratio of 0.5 [17].

**Fig. 5:**
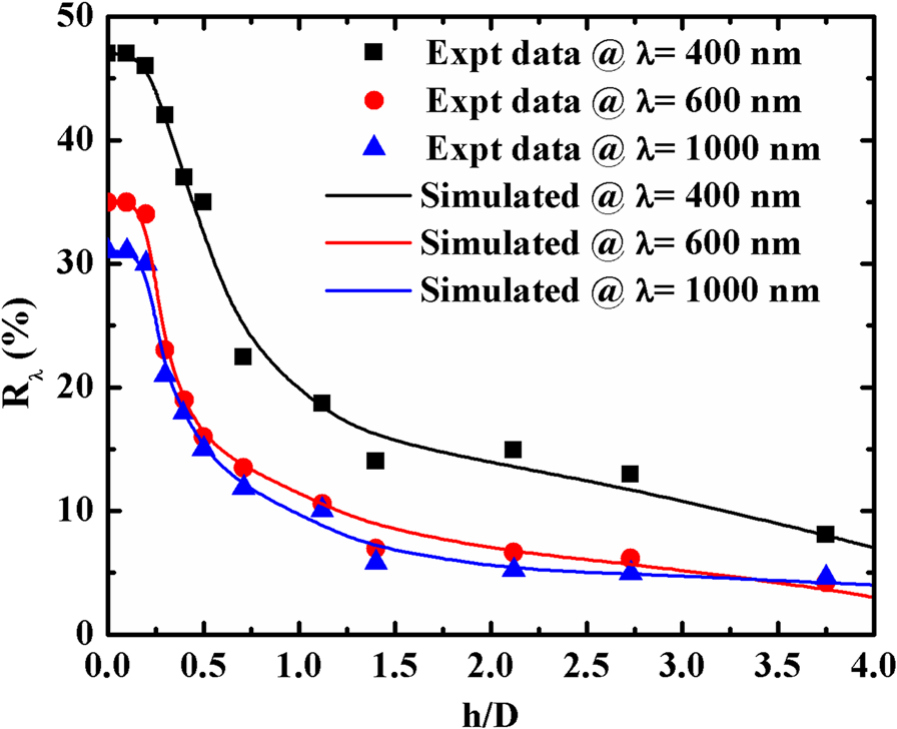
Variation of reflectance values at wavelengths of 400, 600, and 1000 nm with *h/D* ratio

The *R*_*λ*_ values with *h/D* ratios at wavelengths of 400, 600, and 1000 nm are shown in Fig. 5. In the case of the paraboloid texture surface, the *h/D* ratio can increase up to 3.75, whereas, for part of the hemispherical texture surface, the maximum *h/D* ratio is 0.5. For *h/D* = 0.2, the reflectance values of the paraboloid texture are the same as the untextured or textured parts of the hemisphere surface [17] (not shown in Fig. 5). The dimension of the structure depends upon the *h/D* ratio. For higher values of the *h/D* ratio (i.e., 10), the paraboloid texture belongs to micro-wires or nano-wires array. It depends on the size of pits and spacing among the textures.

**Fig. 6:**
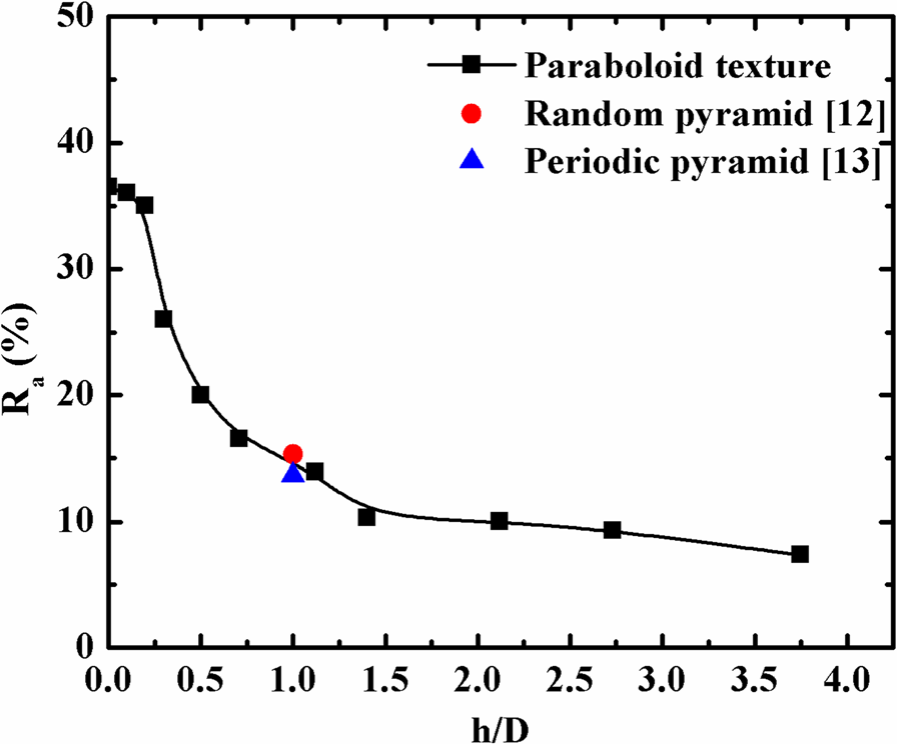
Variation of average reflectance values with *h/D* ratio along with reflectance values of random [12] and regular [13] pyramid texture silicon surfaces

**Fig. 7:**
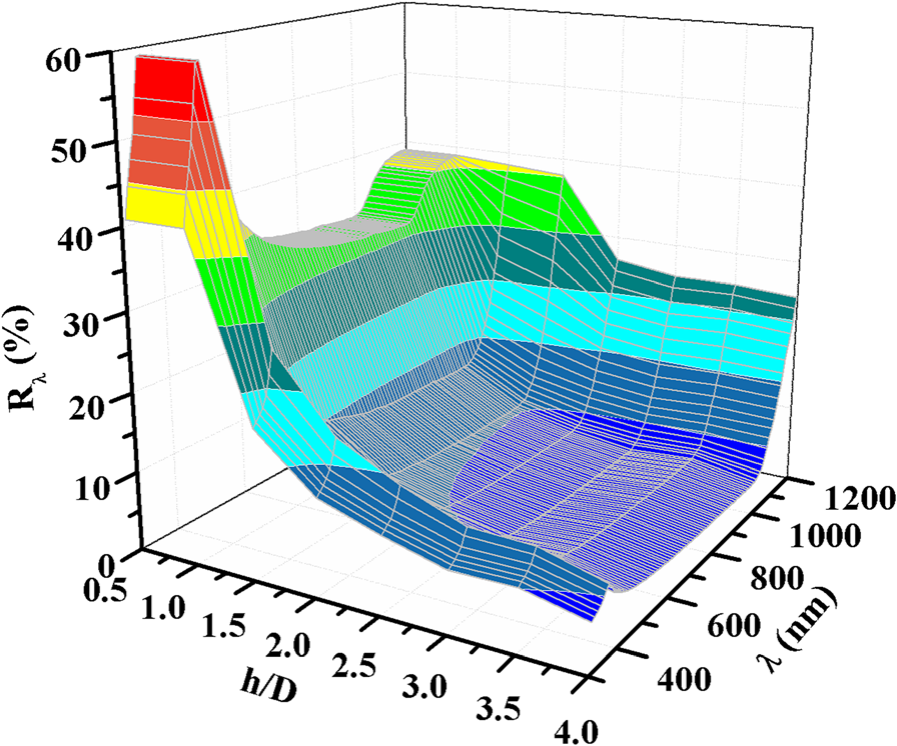
Simulated reflectance as a function of wavelength and *h/D* ratio using the paraboloid model on our texture samples is shown in Fig. 2 using their corresponding spacing values from Table 1

The average reflectance (*R*_a_) values in the wavelength range of 300–1200 nm based on Eq. (17) is shown in Fig. 6.

17 $$ {R}_{\mathrm{a}}=\frac{{\displaystyle {\int}_{\lambda =300}^{1200}{R}_{\lambda }d\lambda }}{{\displaystyle {\int}_{\lambda =300}^{1200}d\lambda }} $$

where *R*_*λ*_ is the reflectance value at wavelength *λ*. Figure 6 also shows the *R*_a_ value of random [12] and regular [13] pyramidal texture silicon surfaces. The reported values of *R*_a_ values of random [12] and regular [13] pyramidal structures in the wavelength range of 300–1200 nm are 15.31 and 13.66, respectively. However, the *R*_a_ value of the acid textured parts of the hemispherical surface in the same wavelength range was found to be ~20 % [17]. It can be seen in Fig. 6 that the *R*_a_ values of paraboloid texture surfaces for the *h/D* ratio more than 1 are lower than the random pyramidal [12] and regular pyramidal [13] silicon surfaces. The *R*_a_, as shown in Fig. 6, which indicates that *R*_a_ is nearly the same for the *h/D* ratio up to 0.2, then falls down sharply for the *h/D* ratio of ~1.5. However, for higher values of the *h/D* ratio, the *R*_a_ values decreases monotonically with a *h/D* ratio up to 3.75. The simulated reflectance as a function of wavelength and *h/D* ratio using the model on our texture samples is shown in Fig. 7. The theoretically predicted and experimentally measured reflectance values in the wavelength range of 500–1200 nm matched well with the *h/D* ratio < 4.

**Fig. 8:**
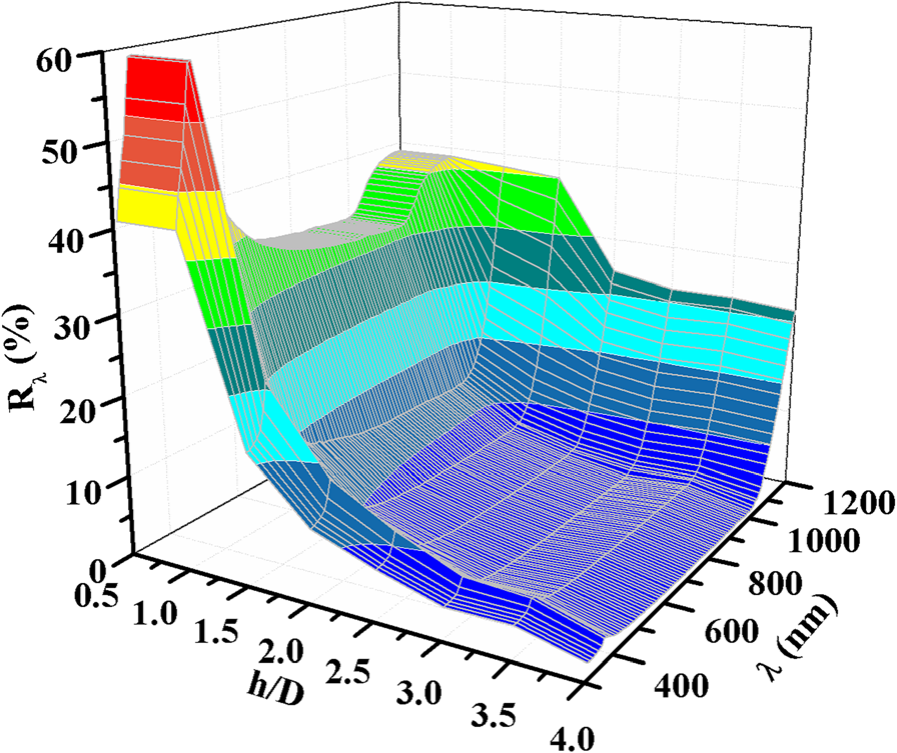
Predicted reflectance values without any space among textures as a function of wavelength and *h/D* ratio using paraboloid model

**Fig. 9:**
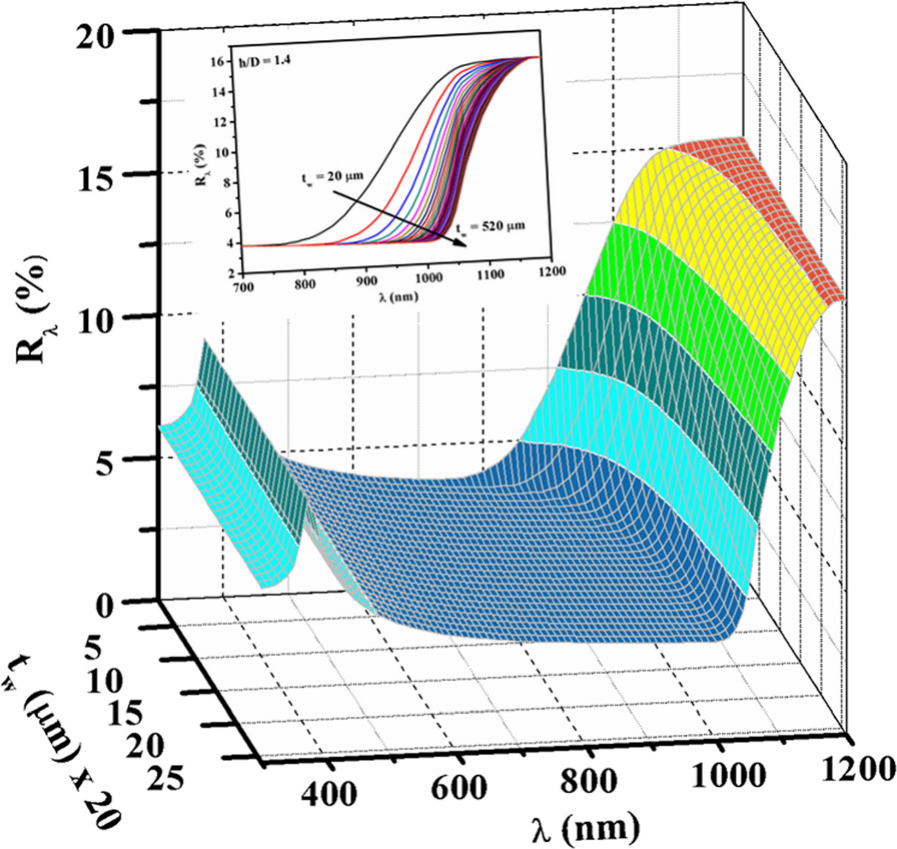
Predicted reflectance values without any space among textures as a function of wavelength and wafer thickness for *h*/*D* = 1.40

The experimental *R*_*λ*_ can be reduced by reducing the *d* values. The predicted *R*_*λ*_ values without any separation among the textures (*d* = 0) as a function of wavelength and *h/D* ratio is shown in Fig. 8. The *R*_*λ*_ also depends on the value of *t*_w_, and it affected the long wavelength region only. The thickness dependent simulated *R*_*λ*_ in the wavelength range of 300–1200 nm for *t*_w_ = 20 to 520 μm with the steps of 20 μm (assuming *d* = 0) is shown in Fig. 9. The dependency of *R*_*λ*_ on *t*_w_ is shown in the inset of Fig. 9. The *R*_*λ*_ is nearly constant up to *λ* ≈ 760 nm with *t*_w_. For high value of *λ*, the *R*_*λ*_ depends on *t*_w_ and increases for higher *λ* values due reflection from back surface. The *λ* value at which *R*_*λ*_ started to increase is shifted towards higher wavelength with the increase of *t*_w_.

After using the passivating layer, the recombination at the surface can be reduced to a very low value. The passivating layer can also work as ARC. Thus, paraboloid structure combined with the passivating layer can be used in PV devices for better performance.

## Conclusions

The paraboloid texture surfaces of Si with different *h/D* ratios were obtained by anisotropic chemical etching using external bias in order to increase the *h/D* ratio. The paraboloid texture surface has a lower reflectance value than the hemispherical, random pyramidal, and regular pyramidal texture surfaces. An optical model was developed to simulate the light trapping and scattering effects of paraboloid Si textured surfaces. The model was experimentally verified by measuring the reflectance values for different *h/D* ratios. The *h/D* ratio of the textured surface was varied up to 3.75. The measured reflectance values matched well with the simulated one in the wavelength of 300–1200 nm. The minimum experimental reflectance value of ~4 % was obtained at a wavelength of 600 nm for the *h/D* ratio of 3.75. Simulation results showed that the reflectance value could be reduced to less than 1 % by reducing the separation among paraboloid textures.
